# Chemical Approaches for Studying the Biology and Pharmacology of Membrane Transporters: The Histidine/Large Amino Acid Transporter SLC7A5 as a Benchmark

**DOI:** 10.3390/molecules26216562

**Published:** 2021-10-29

**Authors:** Mariafrancesca Scalise, Raffaella Scanga, Lara Console, Michele Galluccio, Lorena Pochini, Cesare Indiveri

**Affiliations:** 1 Unit of Biochemistry and Molecular Biotechnology, Department DiBEST (Biologia, Ecologia, Scienze della Terra), University of Calabria, Via P. Bucci 4C, 87036 Arcavacata di Rende, Italy; mariafrancesca.scalise@unical.it (M.S.); raffaella.scanga@unical.it (R.S.); lara.console@unical.it (L.C.); michele.galluccio@unical.it (M.G.); lorena.pochini@unical.it (L.P.); 2 CNR Institute of Biomembranes, Bioenergetics and Molecular Biotechnologies (IBIOM), National Research Council, Via Orabona 4, 70126 Bari, Italy

**Keywords:** membrane transport, protein ligands, cancer, placenta, blood brain barrier, SLC (SoLute Carriers), amino acids

## Abstract

The localization of membrane transporters at the forefront of natural barriers makes these proteins very interesting due to their involvement in the absorption and distribution of nutrients and xenobiotics, including drugs. Over the years, structure/function relationship studies have been performed employing several strategies, including chemical modification of exposed amino acid residues. These approaches are very meaningful when applied to membrane transporters, given that these proteins are characterized by both hydrophobic and hydrophilic domains with a different degree of accessibility to employed chemicals. Besides basic features, the chemical targeting approaches can disclose information useful for pharmacological applications as well. An eminent example of this picture is the histidine/large amino acid transporter SLC7A5, known as LAT1 (Large Amino Acid Transporter 1). This protein is crucial in cell life because it is responsible for mediating the absorption and distribution of essential amino acids in peculiar body districts, such as the blood brain barrier and placenta. Furthermore, LAT1 can recognize a large variety of molecules of pharmacological interest and is also considered a hot target for drugs due to its over-expression in virtually all human cancers. Therefore, it is not surprising that the chemical targeting approach, coupled with bioinformatics, site-directed mutagenesis and transport assays, proved fundamental in describing features of LAT1 such as the substrate binding site, regulatory domains and interactions with drugs that will be discussed in this review. The results on LAT1 can be considered to have general applicability to other transporters linked with human diseases.

## 1. General Aspects of Chemical Approaches on Transporters

Transporters localized at the plasma membrane of all living cells, as well as at the organelle membranes of eukaryotic cells, are crucial for cell life due to the ability of these proteins to mediate the uptake, distribution and excretion of metabolites [1,2]. The relevance of membrane transporters is well recognized nowadays by the scientific community, by pharma and biotech companies [3,4,5]. In the beginning, the study of these proteins has been hampered by their unfavorable intrinsic chemical and physical features, causing a delay in achieving a systematic knowledge that is still far from being completed. The following paragraphs will deal with one of the most widely used strategies to approach the study of membrane transporters, i.e., chemical targeting, which was subjected to continuous developments and improvements over the years [6,7].

### 1.1. Chemical Targeting for Structure/Function Relationships Studies of Membrane Transporters

Chemical targeting of specific amino acid residues has been initially used in whole cells or in isolated organelles to distinguish and recognize the activity of a specific transporter from that of others [8]. In the pre-genomic era, this strategy enabled the first classification of membrane transporters on the basis of the sensitivity towards specific chemicals acting as inhibitors of transport. Then, chemical targeting has been coupled to biotechnology approaches such as heterologous over-expression in microorganisms, site-directed mutagenesis, and in vitro/ex vivo functional assay [7]. This enlarged the number and the appropriateness of the information on membrane transporters, including substrate specificities and the characterization of interactors and regulatory effectors. These results have been achieved by employing a large variety of reagents able to specifically target reactive moieties of amino acid residues, the most common being cysteine and, to a much lesser extent, lysine. In particular, the SH group of cysteine gives rise to different kinds of reactions at physiological pH, ranging from alkylation (maleimides) to the formation of mixed disulfides (methane thiosulphonates) or the formation of metal-S bonds (simple or derivatized heavy metals) [7,9]. It has to be stressed that in the pre-structure era, i.e., before the solution of the first 3D structure of transporters, chemical targeting and site-directed mutagenesis were the only available tools to guess the structural features of a protein and, later on, to prove the suitability of homology models built on the basis of the first 3D structures. Very recently, the scientific revolution driven by CryoEM (Cryo Electron Microscopy) technology greatly increased the number of available 3D structures, enlarging the knowledge on the structural aspects of transporters [10]. This development notwithstanding, the chemical targeting approach is still considered a valid tool for structure and topology validation. As an example, the conformational changes occurring during the transport process cannot be described by a single 3D structure that is only a shot of the entire transport cycle; in this case, chemical targeting helps in describing the dynamics of a specific amino acid residue(s) and/or domain(s) [8]. Moreover, the presence of intrinsically disordered regions and unstructured loops cannot be fully described only by a structural biology approach; therefore, chemical targeting helps in understanding the presence of exposed and, thus, reactive protein residues. It has to be highlighted here that many data earlier obtained via chemical targeting correlate well with the 3D structures, further emphasizing the relevance and the suitability of this experimental approach [11]. One of the membrane proteins that best proves the key role played by the chemical targeting approach for dramatically enlarging knowledge on function, structure and regulation is the fifth member of the SLC7 family, i.e., the amino acid transporter LAT1 (SLC7A5). The dedicated sections summarize the studies conducted on LAT1, which can be considered as a benchmark in the field.

### 1.2. Experimental Methodologies for Applying Chemical Approaches

The use of a chemical approach requires, besides appropriate chemical compounds, a method to evaluate the effects on the transport protein activity upon reversible or covalent reaction with the chemicals. As described in Section 1.1, the most frequently used chemicals are (i) reagents that bind to specific amino acid residues, (ii) compounds that mimic the substrate structure and, thus, interfere with the binding of substrate(s) to the protein(s) of interest. In each case, the most widely-used test employed for validating the action of the chemicals is the measurement of the transport activity and its variations upon interaction with the compounds [12,13]. The activity of a membrane transporter can be measured directly in intact cells using human cell lines in which the transporter is expressed in an active form. Alternatively, an in vitro experimental model can be used consisting of artificial membrane vesicles assembled with phospholipids. This tool mimics cell membrane harbouring the protein of interest that is obtained via heterologous expression in microorganisms or human cells. The assembled experimental system is called proteoliposomes, which has the advantage to eliminate the interferences given by other transporters or enzymes, which, on the contrary, are present in intact cells. The interaction with a chemical can be studied to obtain information on the structure/function relationships of the transporters using both the described experimental models [2]. In most cases, the transport activity inhibition is the result of the interaction of chemicals with the transporter. The first, and raw, observed result is normally a loss of or a decrease in activity. Then, by more detailed investigation, the dose/response analyses are performed from which the IC_50_ value(s) for inhibition can be obtained; these data give information on the efficacy of the effect of the chemical(s) and the affinity of the transporter towards the compound(s). Another very useful piece of information is the type of inhibition that can be derived from inhibition kinetics. From these analyses, a competitive inhibition can be discriminated from a non-competitive inhibition. The first one is typical of substrate-mimicking compounds; the second one is observed in the case of covalent interacting compounds. In any case, the action of a chemical on the transport activity provides unequivocal information on the role of some amino acid residues or specific protein domains on the function of a membrane transporter. Further investigations coupling site-directed mutagenesis to homology models or 3D structures, when available, lead to more precise and detailed information on the structure/function relationships of the protein. These basic studies are essential for applied research, aimed at designing specific molecules to be employed as lead compounds for drug discovery.

## 2. LAT1 as a Benchmark of the Chemical Targeting Approach

This section will describe the main features of human LAT1, the fifth member of the SLC7 family. This transporter is crucial for the metabolism of both normal tissues and human cancers. Indeed, in the last decades, the interest around LAT1 dramatically increased due to several pathological implications caused by expression/function derangements; as an example, LAT1 malfunctioning at the Blood Brain Barrier (BBB) or placenta, is causative of Autism Spectrum Disorders (ASD) or altered foetus development, respectively. Moreover, the over-expression of LAT1 in several human cancers turned pharma companies onto the idea of this protein being a hot target for novel or repurposed drugs. Therefore, LAT1 can now be considered as a benchmark of biochemical studies employing chemical targeting approaches that, coupled to bioinformatics and site-directed mutagenesis, shed light on the structure/function relationships of this protein and opened the way to the design of novel drugs.

### 2.1. Features of the SLC7 Family

**Figure 1:**
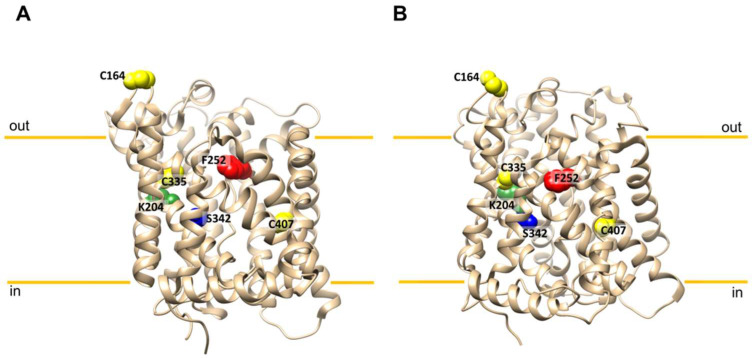
3D structures of hLAT1. (**A**) The cryo-EM structure of hLAT1 in inward conformation (PDB ID: 6IRT, chain B) was represented as ribbon using Chimera v.1.7 software [18]. (**B**) The cryo-EM structure of hLAT1 in inward conformation (PDB ID: 7DSQ, chain B) was represented as ribbon using Chimera v.1.7 software [18]. C164, C335 and C407 residues are highlighted in yellow spheres, F252 is highlighted in a red sphere, S342 is highlighted in a blue sphere and K204 is highlighted in a green sphere. The cell membrane and its orientation are indicated.

The SLC7 family includes 15 members and is part of the larger Amino acid Polyamine organo Cation (APC) superfamily [14,15,16]. Based on their structural and functional features, the SLC7 family members are divided into two groups: Cationic Amino acid Transporters (CATs) and Heterodimeric Amino acid Transporters (HATs). The most striking difference between the two groups resides in their structural features: CATs are single-chain *N*-glycosylated membrane proteins, whereas HATs are formed by two different polypeptide chains, one belonging to the SLC7 family (light chain) and another one belonging to the SLC3 family (heavy, *N*-glycosylated chain). The interaction between the two chains occurs through a disulfide bridge at the level of two conserved cysteine residues (Figure 1); in the case of LAT1 (a SLC7A5-light chain), the link occurs with CD98 (a SLC3A2–heavy chain) that seems to be responsible for the trafficking to the plasma membrane of the entire LAT1/CD98 complex [14,16], as demonstrated in studies conducted in cell-free systems. This shows that LAT1 is the sole transport competent unit while CD98 does not have intrinsic transport function(s) [17].

### 2.2. SLC7A5 Expression and Function: Role in Cell Metabolism

**Figure 2:**
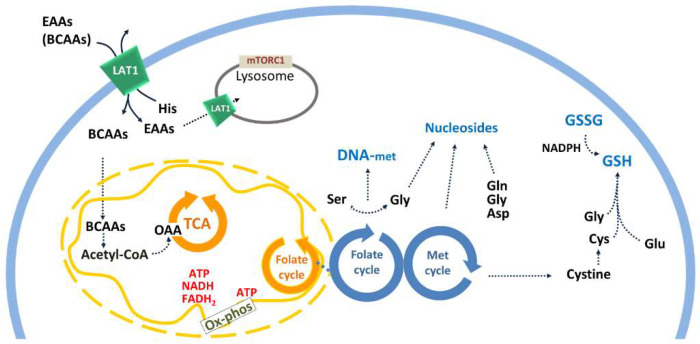
Schematic representation of specific pathways linked to LAT1 expression in cell membranes. LAT1 is represented in green in both plasma and lysosomal membranes. LAT1 mediates the exchange of nine EAAs (Essential Amino Acids), including BCAAs (Branched Chain Amino Acids), from external side of cell membrane, for internal histidine. Intermediates and amino acids (three letter codes) are represented in black; energetic substrates are depicted in red. Transport phenomena are represented by continuous arrows; enzyme pathways are represented by dotted arrows. Mitochondrial or cytosolic cyclic pathways are depicted in orange or blue, respectively. Lysosomes and mTORC1 are indicated in grey. TCA (tricarboxylic acid cycle), OAA (oxaloacetate), DNA-met (DNA methylation), GSH (reduced glutathione), GSSG (oxidized glutathione), ox-phos (oxidative phosphorylation), Met cycle (methionine cycle).

The human gene encoding for LAT1 protein is localized at chromosome 16q24.2 (locus ID8140) and is formed by 10 exons which are spliced into two transcripts. The first variant (NM_003486.6) codes for the LAT1 full length, consisting of 507 amino acids with a theoretical molecular mass of 55 KDa. LAT1 is a ubiquitously expressed protein, even if it is expressed at very low levels; the highest level of expression is observed in testis, bone marrow, brain and placenta, where the protein plays the role of providing tissues with essential amino acids required for cell homeostasis [16,19]. Regarding the subcellular localization, LAT1 is mainly localized at the basolateral membrane of polarized epithelial cells; a peculiar exception is that of BBB and placenta, where LAT1 is localized both at the apical and basolateral membranes. It has been also proposed that LAT1 has a lysosomal localization for mTORC1 activation [20] (Figure 2). Interestingly, the LAT1 expression is strongly increased in several human cancers and those deriving from tissues that normally do not express LAT1 [21,22,23]. This makes LAT1 a hot pharmacological target for newly designed drugs or repurposed old drugs for anticancer therapy. LAT1 function has been studied in different experimental models since the late ‘90s; at first, cell models were employed demonstrating that this protein is responsible for the exchange across the cell membrane of essential amino acids by a Na^+^ and pH-independent mechanism. These transport features have been later demonstrated in cell-free models, i.e., proteoliposomes reconstituted with recombinant human LAT1 (hLAT1), overexpressed in *E. coli*. Initially, this experimental model confirmed that LAT1 is the sole transport competent unit of the heterodimer LAT1/CD98 [17]. Moreover, the functional characterization of LAT1 revealed a peculiar asymmetry towards substrates, with methionine, isoleucine, phenylalanine and leucine being only inwardly transported, whereas tyrosine and histidine are bidirectionally transported. Interestingly, this functional asymmetry overlaps the kinetic asymmetry; indeed, external Km has been measured in the micromolar range, while internal Km is in the millimolar range. In this context, the single-protein approach of proteoliposome was also helpful to decipher the kinetic transport mechanism of LAT1 [24]. Indeed, by performing a bi-substrate analysis, it has been demonstrated that the transport of essential amino acids occurred with a random simultaneous mechanism, i.e., with no preferential binding order of the substrates on the two sides of the transporter. Apart from the mentioned essential amino acids, LAT1 recognizes cysteine and, with a much lower affinity, glutamine. The specificity of LAT1 underlies the physiological function of this protein [19,25]: as an example, the high affinity transport of histidine links LAT1 function to the synthesis of aspartate and glutamate, as well as histamine. This feature makes LAT1 relevant for brain homeostasis and inflammatory response [26,27]. It is noteworthy that the concentration gradient of histidine existing across the plasma membrane furnishes the driving force for the exchange with other essential amino acids [28]. It is important to stress that the presence of LAT1 in BBB and in the placenta, makes this protein fundamental for brain homeostasis and normal fetus development, respectively; indeed, embryos KO for LAT1 are not vital [29]. In this scenario, several studies linked LAT1 to the toxicity exerted by mercury compounds in the brain and placenta. Moreover, the ability of LAT1 to recognize also glutamine has been historically linked to cancer biology; indeed, cancer cells are characterized by the phenomenon known as “glutamine addiction”, due to their increased need for this amino acid employed in energy production and signalling, besides protein synthesis. Following this finding, glutamine is nowadays considered a “conditionally-essential” amino acid [30]. However, the observation that glutamine is a low-affinity LAT1 substrate redirected the role of this protein towards another function in cancer cells and linked it to the much greater efficiency in the transport of essential amino acids [24]. One of the hallmarks of cancer is the tremendous high proliferation rate with a consequent need for “de novo” synthesis of fatty acids and macromolecules, including proteins. Therefore, it is not a surprise that LAT1 became fundamental in providing cells with essential amino acids required to sustain the synthesis processes (Figure 2). It is important to highlight that some amino acids are also required for their ability in regulating different cell pathways responsible for cell homeostasis. A good example is the LAT1 substrate methionine, which is one of the players of the 1C atom metabolism, a complex pathway including the methionine cycle and folate cycle [31]. In this context, methionine acts as a methyl-donor through S-Adenosyl-methionine (SAM) for regulating DNA methylation and, then, epigenetics [32]. Noteworthy, cancer cells are often characterized by aberrant DNA methylation responsible for altered protein expression and, hence, cell growth (Figure 2). Moreover, methionine is also involved in cysteine and H_2_S synthesis, being relevant for redox cell homeostasis. An additional LAT1-substrate is the branched-chain amino acid leucine that is the most abundant amino acid in proteins. This feature makes leucine a key signalling factor for sensing amino acid sufficiency in cells via interaction with mTORC1, the crucial manager in balancing cell life and death. Interestingly, LAT1 was found to be located at the lysosomal membrane as well, the site of action of mTORC1 [20]. Moreover, leucine also has a role in the utilization of glutamine in cancer because it can mediate the allosteric regulation of glutamate dehydrogenase, which is the enzyme converting the glutamine-derived glutamate into α-ketoglutarate [33]. LAT1 is also able to recognize a wide range of substrates alternative to amino acids; as an example, thyroid hormones T3 and T4 are considered substrates of the transporter [34]. Furthermore, LAT1 is involved in the transport of several drugs including the precursor of dopamine l-DOPA (levodopa), melphalan, baclofen and gabapentin [22]. This property attracted the attention of pharmacology research on LAT1, due to its expression in the BBB that is impermeant to virtually all drugs. Therefore, an approach that exploits LAT1 as a vehicle of drugs has been set up over the years, namely the “prodrug approach” [34]. In other words, pharmacological compounds are coupled to amino acids, which are taken up by LAT1 and then released in cells to allow for exerting their actions [35]. Finally, the availability of recombinant hLAT1 over-expressed in *E. coli* allowed for deepening of the study on some regulatory properties of the protein, using the reconstitution in proteoliposomes. As an example, it has been recently demonstrated that cholesterol, included in the vesicle membranes, can increase the transport activity of LAT1 without affecting the affinity for natural substrates [36]. The stimulation of transport activity is due to the physical interaction between cholesterol and LAT1, in line with the recently solved 3D structure of hLAT1-CD98 that showed the presence of lipid densities linked to the heterodimeric complex [37,38]. This finding has been observed also in native cell membranes, where depletion of cholesterol by methyl cyclodextrin, caused a decrease in the transport of l-DOPA [39]. Thanks to the reconstitution in proteoliposomes, another property has been revealed concerning LAT1 biology, i.e., the regulation by intraliposomal (intracellular) ATP. In particular, this nucleotide can stimulate LAT1 transport activity only upon the interaction of the protein with cholesterol, due to a conformational change occurring at the level of a nucleotide binding site in the internal side of the protein [36] (Figure 1).

### 2.3. SLC7A5: Regulation of Expression and Involvement in Diseases

The knowledge on the regulation of LAT1 cell expression, PTMs and trafficking are far from being completed. Notwithstanding, a boost in this field has been given by the discovery that LAT1 is involved in several human diseases. As an example, the involvement of LAT1 in the migration of fibroblast in rheumatoid arthritis promoted the discovery that IL-17, IL-2 and long non-coding RNA can stimulate LAT1 expression [40,41]. Moreover, the decreased expression of LAT1 in pancreatitis explains the simultaneous reduced production of digestive enzymes, whose synthesis requires amino acids transported by LAT1 [42]. The alteration of branched-chain amino acid metabolism in diabetes has been linked to the ability of glucose and insulin to modulate LAT1 expression levels in the pancreas, explaining several symptoms of the disease, such as sarcopenia and damages to the retina [43,44]. Another interesting link between diabetes and LAT1 derives from the role played by LAT1 in the placenta; indeed, maternal obesity and insulin resistance seem to be responsible for upregulation of LAT1 in the placenta, with fetus overgrowth and increased risk for diabetes occurring after birth and in adulthood [45]. Conversely, LAT1 downregulation is linked to a high-risk condition for new-borns, namely Intra-Uterine Growth Restriction (IUGR), [46] characterized by leucine and phenylalanine supply; in turn, this condition is predictive of cardiovascular and metabolic diseases in childhood and adulthood. Moreover, the presence of LAT1 in BBB links this protein to neurodegenerative disorders such as Parkinson’s Disease (PD) [47] and ASD. In particular, PD seems to be linked to an altered distribution of l-DOPA, a well-known LAT1 substrate [48]. Regarding ASD, two naturally occurring LAT1 mutations have been described as responsible for such phenotype due to abnormal accumulation of histidine and, consequently, lowered accumulation of other essential amino acids such as tryptophan, responsible for neurological development [49,50]. Concerning the well-documented expression of LAT1 in human cancers, it has to be stressed that a common molecular mechanism underlying this phenomenon is still lacking, despite the assumption that LAT1 is a hallmark of cancer development and progression. Indeed, several pathways have been indicated as responsible for LAT1 over-expression, including the proto-oncogene c-Myc [51], the YAP (Yes1 Associated Transcriptional Regulator)/TAZ0 transcriptional regulators promoting cell proliferation [52], the hypoxia-inducible factor HIF2α [53], methyltransferase EZH2 responsible for regulating cell differentiation [32]. Notably, LAT1 over-expression is also a prognostic factor of metastasis [51], as well as the over-expression of the ancillary protein CD98 [54]. However, CD98 is a protein with additional roles not related to transporter regulation, ranging from immune system regulation, cell growth activation and cell adhesion to integrin signalling [15,55,56]. In line with these observations, a sizable number of studies focused on designing antibodies against CD98 to counteract cell proliferation and metastasis [57,58].

### 2.4. SLC7A5 Structure/Function Relationships and the Chemical Targeting Approach

It is now well acknowledged that the delay in the studies of structure/function relationships of membrane transporters (with respect to enzymes) is due to the lack or scarcity of refined 3D structures up to a few years ago. After the “resolution revolution” triggered by CryoEM technology [10], a growing number of three-dimensional structures of human transporters has been deposited in the PDB (Protein Data Bank) database. Among these, in 2019 two papers described the 3D structures of LAT1 in different conformations and with interacting ligands. Before that time, the information on the structure/function relationship on the transporter has been collected on the basis of homology structural models obtained from comparison with the bacterial homologue AdiC, a membrane transporter for arginine/agmatine from E. coli, even though the percentage of identity between the two proteins is low [59,60]. Notwithstanding, the key molecular determinant for substrate translocation of LAT1 has been identified, namely the residue F252 (Figure 1), which is the orthologue of a tryptophan residue in the arginine/agmatine transporter AdiC of *E. coli*. In particular, the F252 residue was revealed to be the essential gate of LAT1, as demonstrated by the disruptive effect of the F252A mutation. Indeed, the transport activity of the recombinant protein harbouring the mutation (F252A) was completely abolished in in vitro assays, whereas the conservative mutation F252W, resembling the bacterial counterpart AdiC, resulted in a functional protein with a lower affinity towards histidine with respect to the WT protein [24]. Later on, these results have been confirmed by the 3D structures obtained both in the presence of substrate and of the competitive inhibitor BCH interacting with the residue F252. Intriguingly, BCH was already the best competitive inhibitor of LAT1 in the pre-structure era, used in either cell systems and proteoliposomes to specifically target the substrate binding site of the transporter [22,25,60]. Interestingly, the study on the substrate binding site of human LAT1 took advantage also of a covalent chemical targeting approach, exploiting the presence of 12 cysteine residues in its primary structure. In this context, the LAT1 reactivity towards small, large, hydrophobic or hydrophilic thiol reagents has been investigated [24]. The protein reconstituted in liposomes was strongly inhibited by the mercury compounds HgCl_2_, Methyl-Hg and Ethyl-Hg, as well as by MTSEA and NEM. In line with the interaction of chemical reagents with thiol residues of cysteines, LAT1 inhibition was reversed by the addition of the reducing agent DTE, indicating that, indeed, the reagents act by binding to thiol residues, also confirming previous data collected in intact cells [61]. The described interactions also unveil a plausible molecular mechanism of mercury toxicity for fetus development: on the one hand and in line with the demonstrated reactivity in vitro, LAT1 would be the target of the pollutant(s) [61] with the consequence of lowering essential amino acids entry in foetal circulation [46]; on the other hand, LAT1 has been proposed as a transporter of mercury in complex with amino acids such as cysteine [62]. Moreover, by employing the tool of LAT1 reconstitution in proteoliposomes, a non-competitive type of inhibition has been revealed for mercury compounds in good agreement with the covalent interaction with cysteine residues. In the frame of the chemical targeting approaches, the molecular docking analysis conducted on the homology model of LAT1 predicted the interaction of the substrate histidine with other residues other than the F252, i.e., the C335, the C407 and the S342 forming the substrate binding site of LAT1 [24] (Figure 1). To validate and refine the predictions, site-directed mutants have been produced and their activity tested. More specifically, the residue C335 and S342 revealed fundamental in the substrate recognition by LAT1; indeed, the mutants C335A and S3422, even though functional, showed a Km towards histidine much higher than that of WT, in line with a loss of affinity from the external side of the protein. On the other hand, the mutant C407A showed impairment in internal Km towards histidine, with no effects on the external side of the protein. Very importantly the two residues C335 and C407 are not preferential targets of large SH-reagents; in fact, the mutants C335A, C407A and C335A/C407A remain sensitive to interaction and inhibition exerted by SH-targeting reagents as large mercury compounds and MTS compounds. The sensitivity to the sole Methyl-Hg was slightly decreased in the double mutant, suggesting that only small covalent chemical reagents can reach the substrate binding site. This indicated that C335 and C407 residues cannot be freely targeted by molecules other than the natural substrate(s) because they are buried in the substrate binding pocket that is closed by the F252 residue (Figure 1). Indeed, this large phenylalanine residue can open only upon the interaction of LAT1 with the substrate(s) or chemical analogue(s) such as BCH, but not with molecules that are chemically different from the substrate [32]. From a detailed analysis of the substrate and chemical analogues specificity, the structural properties required for a molecule to be plausibly transported by LAT1 were identified: (i) the presence of vicinal carboxylic and amino groups as demonstrated by the lack of transport of dopamine, serotonin and GABA [50]; (ii) the presence of a large side group, according to the first acronym Large Amino Acid Transporter 1 (LAT1); (iii) the hydrophobicity of the side group. Very interestingly, all the listed features, firstly hypothesized in preliminary studies on rat LAT1 [63] and then confirmed in intact cells and proteoliposomes [17], found the definitive proof when the 3D structure of hLAT1 (Figure 1), in complex with CD98 has been solved [38].The described data highlight that chemical targeting on WT/mutants, together with computational predictions, allowed defining the layout of the LAT1 substrate binding site some years earlier than the resolution of the 3D structure. Still in the frame of the chemical targeting approach is the very recent study of the regulation of LAT1 by the physical interaction with cholesterol [36]. Indeed, the LAT1 transport activity is augmented by cholesterol present in proteoliposomes as well as in native cell membranes. This interaction could not be predicted using bacterial counterparts of LAT1 because cholesterol is not present in the 3D structure of AdiC or other similar proteins; on the contrary, lipid densities have been found in several membrane transporters from a eukaryotic origin in the last years, including dDAT [64] and hSERT [65], which share the same LeuT fold with hLAT1. From the comparison with these structures, some areas for cholesterol binding have been predicted by bioinformatics on LAT1 [36]. In the same study, a nucleotide binding site was predicted, followed by the identification of the key residue, namely K204, which is responsible for recognition of three-phospho-nucleotides, such as ATP. This result was achieved by coupling bioinformatics and site-directed mutagenesis; indeed, the mutation K204Q or K204A triggered a decrease of the stimulatory effect exerted by ATP on LAT1 transport activity [36]. Once again, the solved LAT1 3D structure revealed the presence of lipid densities attributable to cholesterol. Intriguingly and by serendipity, it has been demonstrated that K204 residue is also responsible for modulating the response of LAT1 to pH. This biochemical feature has been disregarded for a long time, with LAT1 transport activity being considered independent of pH; importantly, this characteristic may further explain LAT1 role in cancers, considering that the tumour microenvironment is often characterized by transient pH changes. In the frame of structure/function relationships, the residue K204 is not far from the substrate binding site (Figure 1) as it occurs also for another bacterial homologue, namely GkApcT [66]. On this basis, it was expected that the residue K204 could have some role in the conformational changes of the protein during the transport cycle. In this case, the appropriate use of the chemical targeting approach allowed getting further insights into the dynamic features of the protein. Indeed, covalent and competitive inhibitors were tested on the mutant K204Q: interestingly, besides an affinity variation towards histidine, the mutant displayed a lack of response to the chemicals that target the substrate binding site, both covalent and competitive, indicating that the K204 is indeed involved in conformational changes occurring during translocation and that its substitution leads to substantial alterations of the protein dynamics triggering loss of interaction with molecules even similar to the substrate [36].

### 2.5. SLC7A5: Chemical Targeting Approach for Drug Design

The involvement of LAT1 in several diseases greatly increased the interest of pharmacological research and, in turn, of pharma companies. This holds particularly true in the case of cancer; indeed, searching for specific LAT1 inhibitors able to chemically knock out this protein is one of the main focuses of anticancer drug research [67]. In particular, two main strategies are followed: the design of chemical compounds that act as substrate analogues (competitive inhibitors) or the design of chemical compounds that bind the protein acting as covalent inhibitors (covalent non-competitive inhibitors). The first group of molecules includes substrate-mimicking compounds designed to chemically target the substrate binding site of LAT1, thus impairing its ability to mediate the uptake of essential amino acids in cells. One of the first examples is the amino acid analogue BCH proposed as an anticancer drug [68]. Following this, other ligands were designed as a result of the combined approach of virtual screening of drug libraries and tests in vitro/ex vivo models: phenylalanine and tyrosine analogues [60,69], triiodothyronine (T3) analogues [70], tryptophan analogues [71] and hydroxamic acids conjugated to LAT1 substrates [72]. In these studies, IC50 values ranging from 1 μM to more than 300 μM have been measured together with the evaluation of their efficacy on cell proliferation. Notably, in 2010 a potent competitive inhibitor was designed and proposed for cancer treatment, the tyrosine analogue JPH203, also known as KYT-0353. This molecule has been revealed as a potent inhibitor both in in vitro and in a mouse model of HT-29 tumours (colon cancer) [73,74,75]. Later on, JPH203 has been shown as effective in other cancer types [76,77,78,79]. By using an osteosarcoma cell line, it has been shown that JPH203 activates the mitochondrial pro-apoptotic pathway triggering cell death [78]. Interestingly, in in vitro and in a mouse-transplanted model of HNC, a synergistic effect with metformin has been observed [80]. Finally, it has been recently demonstrated that JPH203 can inhibit the proliferation of anaplastic thyroid cancer (ATC) decreasing the size of xenograft models [81], representing one of the most advanced results in the application of drug design on a membrane transporter for cancer treatment. Even though the strategy of competitive inhibitor design is most commonly used in novel drug design or in repurposing old drugs, a specific pitfall of this approach is the possibility that the presence of natural substrate(s) may displace the drug(s), reducing or eliminating the efficacy and the beneficial effects. Therefore, an alternative strategy is that of covalent drugs. However, there are not very many of these drugs. Very famous examples are two blockbuster drugs, namely acetylsalicylate and penicillin able to react with serine residues of the cyclo-oxygenases and DD-transpeptidase, respectively [82,83]. Concerning membrane transporters as a target of covalent drugs, eminent examples are the purinoceptor P2Y12 and the gastric proton pump, whose cysteine residues are targeted by two commonly used drugs, i.e., clopidogrel and omeprazole (and derivatives), respectively [84,85]. This approach is currently known as targeted covalent inhibitors (TCIs), in which a non-covalent interaction firstly occurs between the drug and the target protein; then, a covalent binding takes place between the electrophilic warhead of the drug and a specific amino acid residue of the target protein [6,86,87]. The main advantage of TCI is that of improving the efficacy, potency and specificity of a designed drug; however, is still an underdeveloped strategy. In this scenario, membrane transporters may represent attractive targets for TCI due to their peculiar location at the boundary between the internal and external environment of a cell and then of an entire tissue. Among amino acids potentially reactive towards drugs, cysteine represents an eminent candidate for drugs based on the TCI approach, given the huge number of different reactions occurring at the level of the thiol group [9,87]. In this respect, LAT1 can be again considered a benchmark for this kind of strategy due to the identification of two cysteine residues in the substrate binding site, as above described (Figure 1). Indeed, a set of 100 compounds with a His-like scaffold able to react with the thiol groups of cysteine residues have been designed. In particular, a library of compounds harbouring a dithiazole group has been synthesized and tested for inhibition using the recombinant human protein reconstituted in proteoliposomes [88]. In this experimental model, a stable and irreversible inhibition with a sub-micromolar IC_50_ value has been measured and the effect on cancer cell death has been assessed. Moreover, by combining the in vitro transport assays and the site-directed mutagenesis, the molecular mechanism of the interaction between the most potent compounds and LAT1 has been investigated. In particular, the cysteine-alanine mutant C407A lost the reactivity towards the dithiazoles compounds demonstrating that the dithiazole scaffold can covalently interact with the substrate binding site of LAT1 triggering an irreversible chemical KO of the protein [88]. Interestingly, dithiazoles have been previously shown to act as anti-fungal, anti-microbial and anti-tumour molecules [89]. Starting from the described strategy, a study employing 1,2,3-triazolyl analogues able to reach the substrate binding site of LAT1 has been recently designed [90]. In a broader view, the chemical targeting approach can be also intended as a prerequisite of drug delivery; in this respect, LAT1 is also an eminent example particularly when thinking about BBB and its impermeability to exogenous substances. This feature is responsible for the inefficacy or low efficacy of several pharmacological treatments. Therefore, as mentioned above, the presence of LAT1 in this body district has been exploited for the “pro-drug” approach, searching for molecules able to be transported by LAT1, complexed to pharmacological compounds with the scope of improving pharmacodynamics of drugs targeting the brain for neurological disorders [35,72,91]. The list of prodrugs based on LAT1 has increased over the years and includes derivatives of ketoprofen, valproate, melphalan and perforin inhibitors [35,91,92,93,94,95]. Finally, targeting LAT1 has been also used for diagnostics and clinics. Indeed, the presence of LAT1 in several human cancers allowed for the design of probes to be employed in diagnostics for improving the potency and specificity of imaging techniques. As an example, LAT1 substrates are used in PET (Positron Emission Tomography) that is a diagnostic technique based on the use of radiolabelled molecules, such as tyrosine, phenylalanine and methionine derivatives, which are delivered specifically to cancer cells via LAT1, allowing for identification of metastasis or tumours already at the early stages of the disease [51]. Another approach is the BNCT Boron neutron capture therapy (BNCT), an anti-cancer therapy based on the irradiation of cancer cells that have been loaded with boron [51,96] and taken up by cancer cells via LAT1 as a phenylalanine-conjugate [96].

## 3. Conclusions

The chemical approach for protein targeting is a long-standing methodology for revealing structure/function relationships of proteins that has been applied more recently to transport systems. The approach is useful to obtain information on structurally unknown proteins as well as to refine the description of molecular mechanisms of transporters whose 3D structure has been solved. The large applicability of the methodology derives from the possibility to target amino acid residues using a large set of chemical reagents that covalently react with specific functional groups of proteins, such as thiol groups of cysteines, but also to target substrate binding sites using non-covalent inhibitors which share structural similarity with the substrates. The great possibility to gain insights on protein functional and structural properties by chemical targeting culminated to the application of this approach for designing specific ligands that exhibit pharmacological activity. One of the most attractive case is that of the essential amino acid transporter LAT1 which is over-expressed in almost all human cancers. Some potent inhibitors have been designed to chemically silence LAT1 in tumours. One of these inhibitors has already reached the clinical trial phase. Thus, the experimentation on LAT1 transporter constitutes a reference study for developing similar strategies suitable for many other transporters linked to human pathology.

## Data Availability

Not applicable.

## References

[1] Cesar-Razquin A., Snijder B., Frappier-Brinton T., Isserlin R., Gyimesi G., Bai X., Reithmeier R.A., Hepworth D., Hediger M.A., Edwards A.M. (2015). A Call for Systematic Research on Solute Carriers. Cell.

[2] Scalise M., Pochini L., Giangregorio N., Tonazzi A., Indiveri C. (2013). Proteoliposomes as tool for assaying membrane transporter functions and interactions with xenobiotics. Pharmaceutics.

[3] Rask-Andersen M., Masuram S., Fredriksson R., Schioth H.B. (2013). Solute carriers as drug targets: Current use, clinical trials and prospective. Mol. Asp. Med..

[4] Lin L., Yee S.W., Kim R.B., Giacomini K.M. (2015). SLC transporters as therapeutic targets: Emerging opportunities. Nat. Rev. Drug Discov..

[5] Nakanishi T., Tamai I. (2011). Solute carrier transporters as targets for drug delivery and pharmacological intervention for chemotherapy. J. Pharm. Sci..

[6] Scalise M., Console L., Galluccio M., Pochini L., Indiveri C. (2020). Chemical Targeting of Membrane Transporters: Insights into Structure/Function Relationships. ACS Omega.

[7] van Iwaarden P.R., Driessen A.J., Konings W.N. (1992). What we can learn from the effects of thiol reagents on transport proteins. Biochim. Biophys. Acta.

[8] Weinglass A.B., Whitelegge J.P., Hu Y., Verner G.E., Faull K.F., Kaback H.R. (2003). Elucidation of substrate binding interactions in a membrane transport protein by mass spectrometry. EMBO J..

[9] Mukherjee H., Grimster N.P. (2018). Beyond cysteine: Recent developments in the area of targeted covalent inhibition. Curr. Opin. Chem. Biol..

[10] Kuhlbrandt W. (2014). Cryo-EM enters a new era. Elife.

[11] deGruyter J.N., Malins L.R., Baran P.S. (2017). Residue-Specific Peptide Modification: A Chemist’s Guide. Biochemistry.

[12] Scalise M., Galluccio M., Pochini L., Console L., Barile M., Giangregorio N., Tonazzi A., Indiveri C. (2017). Studying Interactions of Drugs with Cell Membrane Nutrient Transporters: New Frontiers of Proteoliposome Nanotechnology. Curr. Pharm. Des..

[13] Hediger M.A., Clemencon B., Burrier R.E., Bruford E.A. (2013). The ABCs of membrane transporters in health and disease (SLC series): Introduction. Mol. Asp. Med..

[14] Verrey F., Closs E.I., Wagner C.A., Palacin M., Endou H., Kanai Y. (2004). CATs and HATs: The SLC7 family of amino acid transporters. Pflug. Arch..

[15] Palacin M., Kanai Y. (2004). The ancillary proteins of HATs: SLC3 family of amino acid transporters. Pflug. Arch..

[16] Fotiadis D., Kanai Y., Palacin M. (2013). The SLC3 and SLC7 families of amino acid transporters. Mol. Asp. Med..

[17] Napolitano L., Scalise M., Galluccio M., Pochini L., Albanese L.M., Indiveri C. (2015). LAT1 is the transport competent unit of the LAT1/CD98 heterodimeric amino acid transporter. Int. J. Biochem. Cell. Biol..

[18] Pettersen E.F., Goddard T.D., Huang C.C., Meng E.C., Couch G.S., Croll T.I., Morris J.H., Ferrin T.E. (2021). UCSF ChimeraX: Structure visualization for researchers, educators, and developers. Protein Sci..

[19] Prasad P.D., Wang H., Huang W., Kekuda R., Rajan D.P., Leibach F.H., Ganapathy V. (1999). Human LAT1, a subunit of system L amino acid transporter: Molecular cloning and transport function. Biochem. Biophys. Res. Commun..

[20] Milkereit R., Persaud A., Vanoaica L., Guetg A., Verrey F., Rotin D. (2015). LAPTM4b recruits the LAT1-4F2hc Leu transporter to lysosomes and promotes mTORC1 activation. Nat. Commun..

[21] Bhutia Y.D., Ganapathy V. (2016). Glutamine transporters in mammalian cells and their functions in physiology and cancer. Biochim. Biophys. Acta.

[22] Scalise M., Galluccio M., Console L., Pochini L., Indiveri C. (2018). The Human SLC7A5 (LAT1): The Intriguing Histidine/Large Neutral Amino Acid Transporter and Its Relevance to Human Health. Front. Chem..

[23] Fuchs B.C., Bode B.P. (2005). Amino acid transporters ASCT2 and LAT1 in cancer: Partners in crime?. Semin. Cancer. Biol..

[24] Napolitano L., Galluccio M., Scalise M., Parravicini C., Palazzolo L., Eberini I., Indiveri C. (2017). Novel insights into the transport mechanism of the human amino acid transporter LAT1 (SLC7A5). Probing critical residues for substrate translocation. Biochim. Biophys. Acta. Gen. Subj..

[25] Mastroberardino L., Spindler B., Pfeiffer R., Skelly P.J., Loffing J., Shoemaker C.B., Verrey F. (1998). Amino-acid transport by heterodimers of 4F2hc/CD98 and members of a permease family. Nature.

[26] Hasegawa S., Ichiyama T., Sonaka I., Ohsaki A., Okada S., Wakiguchi H., Kudo K., Kittaka S., Hara M., Furukawa S. (2012). Cysteine, histidine and glycine exhibit anti-inflammatory effects in human coronary arterial endothelial cells. Clin. Exp. Immunol..

[27] Sasahara I., Fujimura N., Nozawa Y., Furuhata Y., Sato H. (2015). The effect of histidine on mental fatigue and cognitive performance in subjects with high fatigue and sleep disruption scores. Physiol. Behav..

[28] Schmid G., Fricke L., Lange H.W., Heidland A. (1977). Intracellular histidine content of various tissues (brain, striated muscle and liver) in experimental chronic renal failure. Klin. Wochenschr..

[29] Ohgaki R., Ohmori T., Hara S., Nakagomi S., Kanai-Azuma M., Kaneda-Nakashima K., Okuda S., Nagamori S., Kanai Y. (2017). Essential Roles of l-type Amino Acid Transporter 1 in Syncytiotrophoblast Development by Presenting Fusogenic 4F2hc. Mol. Cell. Biol..

[30] Vander Heiden M.G., Cantley L.C., Thompson C.B. (2009). Understanding the Warburg effect: The metabolic requirements of cell proliferation. Science.

[31] Sanderson S.M., Gao X., Dai Z., Locasale J.W. (2019). Methionine metabolism in health and cancer: A nexus of diet and precision medicine. Nat. Rev. Cancer.

[32] Dann S.G., Ryskin M., Barsotti A.M., Golas J., Shi C., Miranda M., Hosselet C., Lemon L., Lucas J., Karnoub M. (2015). Reciprocal regulation of amino acid import and epigenetic state through Lat1 and EZH2. EMBO J..

[33] Scalise M., Pochini L., Galluccio M., Console L., Indiveri C. (2017). Glutamine Transport and Mitochondrial Metabolism in Cancer Cell Growth. Front. Oncol..

[34] del Amo E.M., Urtti A., Yliperttula M. (2008). Pharmacokinetic role of l-type amino acid transporters LAT1 and LAT2. Eur. J. Pharm. Sci..

[35] Puris E., Gynther M., Huttunen J., Petsalo A., Huttunen K.M. (2017). l-type amino acid transporter 1 utilizing prodrugs: How to achieve effective brain delivery and low systemic exposure of drugs. J. Control. Release.

[36] Cosco J., Scalise M., Colas C., Galluccio M., Martini R., Rovella F., Mazza T., Ecker G.F., Indiveri C. (2020). ATP modulates SLC7A5 (LAT1) synergistically with cholesterol. Sci. Rep..

[37] Yan R., Zhao X., Lei J., Zhou Q. (2019). Structure of the human LAT1-4F2hc heteromeric amino acid transporter complex. Nature.

[38] Lee Y., Wiriyasermkul P., Jin C., Quan L., Ohgaki R., Okuda S., Kusakizako T., Nishizawa T., Oda K., Ishitani R. (2019). Cryo-EM structure of the human l-type amino acid transporter 1 in complex with glycoprotein CD98hc. Nat. Struct. Mol. Biol..

[39] Dickens D., Chiduza G.N., Wright G.S., Pirmohamed M., Antonyuk S.V., Hasnain S.S. (2017). Modulation of LAT1 (SLC7A5) transporter activity and stability by membrane cholesterol. Sci. Rep..

[40] Sinclair L.V., Rolf J., Emslie E., Shi Y.B., Taylor P.M., Cantrell D.A. (2013). Control of amino-acid transport by antigen receptors coordinates the metabolic reprogramming essential for T cell differentiation. Nat. Immunol..

[41] Yu Z., Lin W., Rui Z., Jihong P. (2018). Fibroblast-like synoviocyte migration is enhanced by IL-17-mediated overexpression of l-type amino acid transporter 1 (LAT1) via the mTOR/4E-BP1 pathway. Amino Acids.

[42] Rooman I., Lutz C., Pinho A.V., Huggel K., Reding T., Lahoutte T., Verrey F., Graf R., Camargo S.M. (2013). Amino acid transporters expression in acinar cells is changed during acute pancreatitis. Pancreatology.

[43] Yamamoto Y., Sawa R., Wake I., Morimoto A., Okimura Y. (2017). Glucose-mediated inactivation of AMP-activated protein kinase reduces the levels of l-type amino acid transporter 1 mRNA in C2C12 cells. Nutr. Res..

[44] Matsuyama R., Tomi M., Akanuma S., Tabuchi A., Kubo Y., Tachikawa M., Hosoya K. (2012). Up-regulation of l-type amino acid transporter 1 (LAT1) in cultured rat retinal capillary endothelial cells in response to glucose deprivation. Drug Metab. Pharm..

[45] Rosario F.J., Kanai Y., Powell T.L., Jansson T. (2015). Increased placental nutrient transport in a novel mouse model of maternal obesity with fetal overgrowth. Obesity (Silver Spring).

[46] Pantham P., Rosario F.J., Weintraub S.T., Nathanielsz P.W., Powell T.L., Li C., Jansson T. (2016). Down-Regulation of Placental Transport of Amino Acids Precedes the Development of Intrauterine Growth Restriction in Maternal Nutrient Restricted Baboons. Biol. Reprod..

[47] Ohtsuki S., Yamaguchi H., Kang Y.S., Hori S., Terasaki T. (2010). Reduction of l-type amino acid transporter 1 mRNA expression in brain capillaries in a mouse model of Parkinson’s disease. Biol. Pharm. Bull..

[48] Kageyama T., Nakamura M., Matsuo A., Yamasaki Y., Takakura Y., Hashida M., Kanai Y., Naito M., Tsuruo T., Minato N. (2000). The 4F2hc/LAT1 complex transports L-DOPA across the blood-brain barrier. Brain. Res..

[49] Asor E., Stempler S., Avital A., Klein E., Ruppin E., Ben-Shachar D. (2015). The role of branched chain amino acid and tryptophan metabolism in rat’s behavioral diversity: Intertwined peripheral and brain effects. Eur. Neuropsychopharmacol..

[50] Tarlungeanu D.C., Deliu E., Dotter C.P., Kara M., Janiesch P.C., Scalise M., Galluccio M., Tesulov M., Morelli E., Sonmez F.M. (2016). Impaired Amino Acid Transport at the Blood Brain Barrier Is a Cause of Autism Spectrum Disorder. Cell.

[51] Hayashi K., Anzai N. (2017). Novel therapeutic approaches targeting l-type amino acid transporters for cancer treatment. World J. Gastrointest. Oncol..

[52] Hansen C.G., Ng Y.L., Lam W.L., Plouffe S.W., Guan K.L. (2015). The Hippo pathway effectors YAP and TAZ promote cell growth by modulating amino acid signaling to mTORC1. Cell Res..

[53] Elorza A., Soro-Arnaiz I., Melendez-Rodriguez F., Rodriguez-Vaello V., Marsboom G., de Carcer G., Acosta-Iborra B., Albacete-Albacete L., Ordonez A., Serrano-Oviedo L. (2012). HIF2alpha acts as an mTORC1 activator through the amino acid carrier SLC7A5. Mol. Cell.

[54] Shin G., Kang T.W., Yang S., Baek S.J., Jeong Y.S., Kim S.Y. (2011). GENT: Gene expression database of normal and tumor tissues. Cancer Inf..

[55] Chillaron J., Roca R., Valencia A., Zorzano A., Palacin M. (2001). Heteromeric amino acid transporters: Biochemistry, genetics, and physiology. Am. J. Physiol. Ren. Physiol..

[56] Cantor J.M., Ginsberg M.H. (2012). CD98 at the crossroads of adaptive immunity and cancer. J. Cell Sci..

[57] Behrens C.R., Ha E.H., Chinn L.L., Bowers S., Probst G., Fitch-Bruhns M., Monteon J., Valdiosera A., Bermudez A., Liao-Chan S. (2015). Antibody-Drug Conjugates (ADCs) Derived from Interchain Cysteine Cross-Linking Demonstrate Improved Homogeneity and Other Pharmacological Properties over Conventional Heterogeneous ADCs. Mol. Pharm..

[58] Hayes G.M., Chinn L., Cantor J.M., Cairns B., Levashova Z., Tran H., Velilla T., Duey D., Lippincott J., Zachwieja J. (2015). Antitumor activity of an anti-CD98 antibody. Int. J. Cancer..

[59] Ilgu H., Jeckelmann J.M., Gapsys V., Ucurum Z., de Groot B.L., Fotiadis D. (2016). Insights into the molecular basis for substrate binding and specificity of the wild-type L-arginine/agmatine antiporter AdiC. Proc. Natl. Acad. Sci. USA.

[60] Geier E.G., Schlessinger A., Fan H., Gable J.E., Irwin J.J., Sali A., Giacomini K.M. (2013). Structure-based ligand discovery for the Large-neutral Amino Acid Transporter 1, LAT-1. Proc. Natl. Acad. Sci. USA.

[61] Boado R.J., Li J.Y., Chu C., Ogoshi F., Wise P., Pardridge W.M. (2005). Site-directed mutagenesis of cysteine residues of large neutral amino acid transporter LAT1. Biochim. Biophys. Acta.

[62] Bridges C.C., Zalups R.K. (2017). Mechanisms involved in the transport of mercuric ions in target tissues. Arch. Toxicol..

[63] Uchino H., Kanai Y., Kim D.K., Wempe M.F., Chairoungdua A., Morimoto E., Anders M.W., Endou H. (2002). Transport of amino acid-related compounds mediated by l-type amino acid transporter 1 (LAT1): Insights into the mechanisms of substrate recognition. Mol. Pharmacol..

[64] Penmatsa A., Wang K.H., Gouaux E. (2013). X-ray structure of dopamine transporter elucidates antidepressant mechanism. Nature.

[65] Laursen L., Severinsen K., Kristensen K.B., Periole X., Overby M., Muller H.K., Schiott B., Sinning S. (2018). Cholesterol binding to a conserved site modulates the conformation, pharmacology, and transport kinetics of the human serotonin transporter. J. Biol. Chem..

[66] Jungnickel K.E.J., Parker J.L., Newstead S. (2018). Structural basis for amino acid transport by the CAT family of SLC7 transporters. Nat. Commun..

[67] Kanai Y. (2021). Amino acid transporter LAT1 (SLC7A5) as a molecular target for cancer diagnosis and therapeutics. Pharm. Ther..

[68] Kaji M., Kabir-Salmani M., Anzai N., Jin C.J., Akimoto Y., Horita A., Sakamoto A., Kanai Y., Sakurai H., Iwashita M. (2010). Properties of l-type amino acid transporter 1 in epidermal ovarian cancer. Int. J. Gynecol. Cancer.

[69] Augustyn E., Finke K., Zur A.A., Hansen L., Heeren N., Chien H.C., Lin L., Giacomini K.M., Colas C., Schlessinger A. (2016). LAT-1 activity of meta-substituted phenylalanine and tyrosine analogs. Bioorg. Med. Chem. Lett..

[70] Kongpracha P., Nagamori S., Wiriyasermkul P., Tanaka Y., Kaneda K., Okuda S., Ohgaki R., Kanai Y. (2017). Structure-activity relationship of a novel series of inhibitors for cancer type transporter l-type amino acid transporter 1 (LAT1). J. Pharm. Sci..

[71] Ylikangas H., Malmioja K., Peura L., Gynther M., Nwachukwu E.O., Leppanen J., Laine K., Rautio J., Lahtela-Kakkonen M., Huttunen K.M. (2014). Quantitative insight into the design of compounds recognized by the l-type amino acid transporter 1 (LAT1). ChemMedChem.

[72] Zur A.A., Chien H.C., Augustyn E., Flint A., Heeren N., Finke K., Hernandez C., Hansen L., Miller S., Lin L. (2016). LAT1 activity of carboxylic acid bioisosteres: Evaluation of hydroxamic acids as substrates. Bioorg. Med. Chem. Lett..

[73] Oda K., Hosoda N., Endo H., Saito K., Tsujihara K., Yamamura M., Sakata T., Anzai N., Wempe M.F., Kanai Y. (2010). l-type amino acid transporter 1 inhibitors inhibit tumor cell growth. Cancer Sci..

[74] Wempe M.F., Rice P.J., Lightner J.W., Jutabha P., Hayashi M., Anzai N., Wakui S., Kusuhara H., Sugiyama Y., Endou H. (2012). Metabolism and pharmacokinetic studies of JPH203, an L-amino acid transporter 1 (LAT1) selective compound. Drug Metab. Pharm..

[75] Toyoshima J., Kusuhara H., Wempe M.F., Endou H., Sugiyama Y. (2013). Investigation of the role of transporters on the hepatic elimination of an LAT1 selective inhibitor JPH203. J. Pharm. Sci..

[76] Rosilio C., Nebout M., Imbert V., Griessinger E., Neffati Z., Benadiba J., Hagenbeek T., Spits H., Reverso J., Ambrosetti D. (2015). l-type amino-acid transporter 1 (LAT1): A therapeutic target supporting growth and survival of T-cell lymphoblastic lymphoma/T-cell acute lymphoblastic leukemia. Leukemia.

[77] Hayashi K., Jutabha P., Maeda S., Supak Y., Ouchi M., Endou H., Fujita T., Chida M., Anzai N. (2016). LAT1 acts as a crucial transporter of amino acids in human thymic carcinoma cells. J. Pharm. Sci..

[78] Choi D.W., Kim D.K., Kanai Y., Wempe M.F., Endou H., Kim J.K. (2017). JPH203, a selective l-type amino acid transporter 1 inhibitor, induces mitochondria-dependent apoptosis in Saos2 human osteosarcoma cells. Korean J. Physiol. Pharm..

[79] Yothaisong S., Dokduang H., Anzai N., Hayashi K., Namwat N., Yongvanit P., Sangkhamanon S., Jutabha P., Endou H., Loilome W. (2017). Inhibition of l-type amino acid transporter 1 activity as a new therapeutic target for cholangiocarcinoma treatment. Tumour Biol..

[80] Ueno S., Kimura T., Yamaga T., Kawada A., Ochiai T., Endou H., Sakurai H. (2016). Metformin enhances anti-tumor effect of l-type amino acid transporter 1 (LAT1) inhibitor. J. Pharm. Sci..

[81] Enomoto K., Sato F., Tamagawa S., Gunduz M., Onoda N., Uchino S., Muragaki Y., Hotomi M. (2019). A novel therapeutic approach for anaplastic thyroid cancer through inhibition of LAT1. Sci. Rep..

[82] Warner T.D., Mitchell J.A. (2002). Cyclooxygenase-3 (COX-3): Filling in the gaps toward a COX continuum?. Proc. Natl. Acad. Sci. USA.

[83] Wright P.M., Seiple I.B., Myers A.G. (2014). The evolving role of chemical synthesis in antibacterial drug discovery. Angew. Chem. Int. Ed. Engl..

[84] Olbe L., Carlsson E., Lindberg P. (2003). A proton-pump inhibitor expedition: The case histories of omeprazole and esomeprazole. Nat. Rev. Drug Discov..

[85] Savi P., Pereillo J.M., Uzabiaga M.F., Combalbert J., Picard C., Maffrand J.P., Pascal M., Herbert J.M. (2000). Identification and biological activity of the active metabolite of clopidogrel. Thromb. Haemost..

[86] Gunnoo S.B., Madder A. (2016). Chemical Protein Modification through Cysteine. Chembiochem.

[87] Scalise M., Console L., Galluccio M., Pochini L., Tonazzi A., Giangregorio N., Indiveri C. (2019). Exploiting Cysteine Residues of SLC Membrane Transporters as Targets for Drugs. Slas. Discov..

[88] Napolitano L., Scalise M., Koyioni M., Koutentis P., Catto M., Eberini I., Parravicini C., Palazzolo L., Pisani L., Galluccio M. (2017). Potent inhibitors of human LAT1 (SLC7A5) transporter based on dithiazole and dithiazine compounds for development of anticancer drugs. Biochem. Pharm..

[89] Konstantinova L.S., Bol’shakov O.I., Obruchnikova N.V., Laborie H., Tanga A., Sopena V., Lanneluc I., Picot L., Sable S., Thiery V. (2009). One-pot synthesis of 5-phenylimino, 5-thieno or 5-oxo-1,2,3-dithiazoles and evaluation of their antimicrobial and antitumor activity. Bioorg. Med. Chem. Lett..

[90] Hall C., Wolfe H., Wells A., Chien H.C., Colas C., Schlessinger A., Giacomini K.M., Thomas A.A. (2019). l-type amino acid transporter 1 activity of 1,2,3-triazolyl analogs of l-histidine and l-tryptophan. Bioorg. Med. Chem. Lett..

[91] Peura L., Malmioja K., Laine K., Leppanen J., Gynther M., Isotalo A., Rautio J. (2011). Large amino acid transporter 1 (LAT1) prodrugs of valproic acid: New prodrug design ideas for central nervous system delivery. Mol. Pharm..

[92] Gynther M., Jalkanen A., Lehtonen M., Forsberg M., Laine K., Ropponen J., Leppanen J., Knuuti J., Rautio J. (2010). Brain uptake of ketoprofen-lysine prodrug in rats. Int. J. Pharm..

[93] Gynther M., Laine K., Ropponen J., Leppanen J., Mannila A., Nevalainen T., Savolainen J., Jarvinen T., Rautio J. (2008). Large neutral amino acid transporter enables brain drug delivery via prodrugs. J. Med. Chem..

[94] Tampio J., Markowicz-Piasecka M., Huttunen K.M. (2021). Hemocompatible l-type amino acid transporter 1 (LAT1)-Utilizing prodrugs of perforin inhibitors can accumulate into the pancreas and alleviate inflammation-induced apoptosis. Chem. Biol. Interact..

[95] Huttunen K.M., Huttunen J., Aufderhaar I., Gynther M., Denny W.A., Spicer J.A. (2016). l-type amino acid transporter 1 (lat1)-mediated targeted delivery of perforin inhibitors. Int. J. Pharm..

[96] Wongthai P., Hagiwara K., Miyoshi Y., Wiriyasermkul P., Wei L., Ohgaki R., Kato I., Hamase K., Nagamori S., Kanai Y. (2015). Boronophenylalanine, a boron delivery agent for boron neutron capture therapy, is transported by ATB^0,+^, LAT1 and LAT2. Cancer Sci..

